# A comparison of vitamin D and cathelicidin (LL-37) levels between patients with active TB and their healthy contacts in a high HIV prevalence setting: a prospective descriptive study

**DOI:** 10.1093/trstmh/trab126

**Published:** 2021-08-16

**Authors:** Patrick Saili Lungu, William Kilembe, Shabir Lakhi, Thomas Sukwa, Evarist Njelesani, Alimuddin I Zumla, Peter Mwaba

**Affiliations:** University of Zambia, School of Medicine, Department Internal Medicine, Lusaka, Zambia; Rwanda Zamba HIV Research Group, Emory University, Lusaka, Zambia; University of Zambia, School of Medicine, Department Internal Medicine, Lusaka, Zambia; Lusaka Apex Medical University, Department of Public Health and Research, Lusaka, Zambia; Lusaka Apex Medical University, Faculty of Medicine, Lusaka, Zambia; Division of Infection and Immunity, University College London, and National Institutes of Health and Research Biomedical Research Centre, University College London Hospitals NHS Foundation Trust, London; Lusaka Apex Medical University, Faculty of Medicine, Lusaka, Zambia

**Keywords:** Active TB, Cathelicidin, Healthy contacts, Vitamin D

## Abstract

**Background:**

Studies from Asia and Europe indicate an association between vitamin D deficiency and susceptibility to TB. We performed an observational case-control study to determine vitamin D and cathelicidin (LL-37) levels and their association with active TB in newly diagnosed and microbiologically confirmed adult TB patients in Zambia, a high HIV prevalence setting.

**Methods:**

Both total vitamin D and LL-37 were measured using ELISA from serum and supernatant isolated from cultured whole blood that was stimulated with heat-killed *Mycobacterium tuberculosis*. Statistical analysis was performed using STATA statistical software version 12.

**Results:**

The median vitamin D in TB patients and healthy contacts was 28.7 (19.88–38.64) and 40.8 (31.2–49.44) ng/ml, respectively (p<0.001). The median LL-37 in TB patients compared with healthy contacts was 1.87 (2.74–8.93) and 6.73 (5.6–9.58) ng/ml, respectively (p=0.0149). Vitamin D correlation with LL-37 in healthy contacts was R^2^=0.7 (95% CI 0.566 to 0.944), p<0.0001. Normal vitamin D significantly predicted a healthy status (OR 4.06, p=0.002).

**Conclusions:**

Significantly lower levels of vitamin D and LL-37 are seen in adults with newly diagnosed active TB. Longitudinal studies across various geographical regions are required to accurately define the roles of vitamin D and LL-37 in preventive and TB treatment outcomes.

## Introduction

Vitamin D (25-OH vitamin D) is a fat-soluble vitamin that appears to play an important role in innate and acquired cellular immune responses to human infection with members of the *Mycobacterium tuberculosis* complex. Both ex vivo and in vivo studies have indicated that vitamin D may enhance the clearance of *M. tuberculosis*.^1-5^ Vitamin D stimulates macrophage production of a pore-forming peptide molecule called cathelicidin (LL-37) and plays an important role in effecting phagocytosis of *M. tuberculosis*. LL-37 is raised in patients infected with intracellular pathogens, including active TB disease.^6^ Torres-Juarez et al. demonstrated the immune modulatory effect of exogenous LL-37 by decreasing the production of TNF-alpha and IL-17 and inducing the production of IL-10, which is anti-inflamatory.^7^ The regions of the world with the highest burden of TB mirror those with the highest burden of vitamin D deficiency. Studies from Asia and Europe have shown an association between vitamin D deficiency and susceptibility to TB.^8-10^ Conventional assumptions that vitamin D deficiency in sub-Saharan Africa may not be an important factor in TB patients require further study. We performed a prospective observational case-control study to determine levels of vitamin D and LL-37 in Zambian adult patients with TB and compare them with healthy controls.

## Materials and Methods

The study was conducted at the University Teaching Hospital located in Lusaka, Zambia. The HIV prevalence in Lusaka stands at 15.1%.

**Figure 1.**
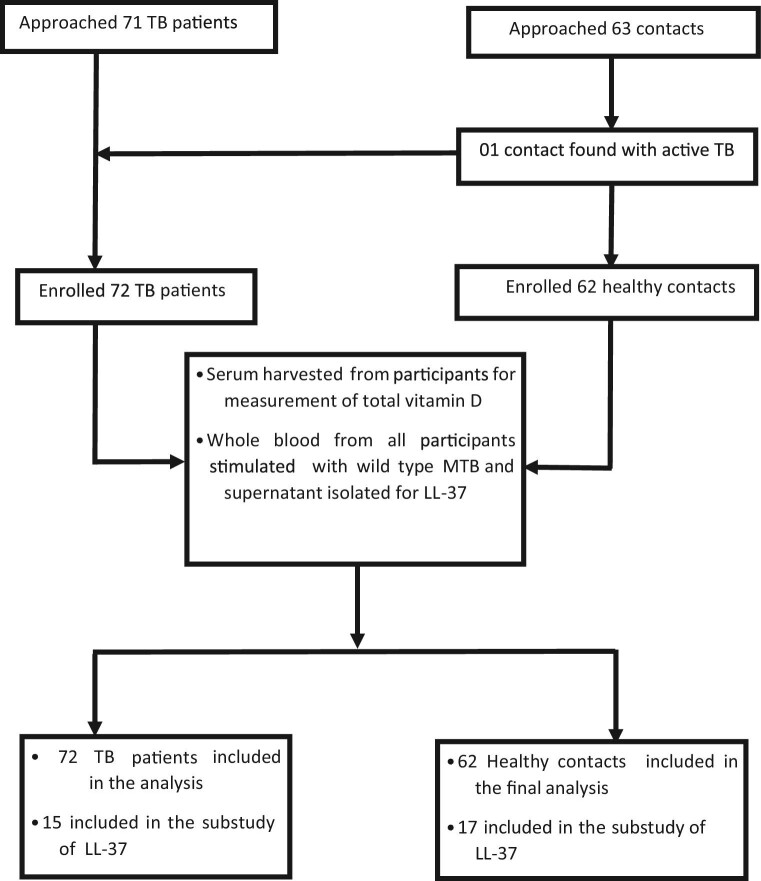
Study flowchart.

After providing informed consent, patients with newly diagnosed active TB (microbiologically confirmed) and their TB-negative, apparently healthy contacts were enrolled into the study. Healthy contacts were asymptomatic, had normal chest X-rays and a negative GeneXpert result on induced sputum. All HIV-positive participants were on efavirenz-based antiretroviral treatment. Patient biodata were collected. All study subjects had an HIV test performed. Whole blood samples were collected prior to the commencement of TB treatment (Figure 1).

### Vitamin D measurement

Total vitamin D (25-OH vitamin D) levels in serum were measured using a Cobas e411 analyser and automated platform that uses the competition principle of ELISA. We used reagents from Roche Diagnostics GmbH (Mannheim, Germany). The process of measuring the vitamin D total takes the following steps in an automated fashion: in the first incubation, 20 μL from each sample is pretreated with dithiothreitol and sodium hydroxide as reagents 1 and 2, respectively; bound 25-hydroxyvitamin D is released from the vitamin D binding protein (VDBP).

In the second incubation, the pretreated sample is labelled with the ruthenium; this forms a complex between the 25-hydroxyvitamin D and the ruthenylated VDBP. A specific unlabelled antibody binds to the 24,25-dihydroxyvitamin D present in the sample and inhibits cross-reactivity to this vitamin D metabolite.

In the third incubation, after adding streptavidin-coated microparticles are added and 25-hydroxyvitamin D labelled with biotin, unbound ruthenylated labelled vitamin D binding proteins, becomes occupied. A complex consisting of the ruthenylated vitamin D binding protein and the biotinylated 25-hydroxyvitamin D is formed and becomes bound to the solid phase via the interaction of biotin and streptavidin. The reaction mixture is aspirated into the measuring cell where the microparticles are magnetically captured on the surface of the electrode. Unbound substances are then removed with ProCell/ProCell M. Application of a voltage to the electrode then induces chemiluminescent emission that is measured by a photomultiplier.

Finally, the results are determined via a calibration curve, which is generated by two-point calibration.^11^ Vitamin deficiency was defined as serum levels of ≤20, insufficiency as 21–29 and normal as 30–100 ng/ml.^11^

### Measurement of LL-37

We used the supernatant materials and from cultured whole blood that was stimulated with heat-killed *M. tuberculosis* and the human LL-37 ELISA kit (HK321-02) produced by Hycult-Biotech (Uden, The Netherlands), with ELISA performed as per the manufacturer's instructions.^12^ Next, 100 μL in duplicate of the standards and samples were transferred into the appropriate or designated wells. A cover was applied and tapped to eliminate air bubbles. The plate was then incubated for 1 h at room temperature. This was followed by washing of the plate four times with a wash buffer solution. Then 100 μL of a tracer was added postwashing to each well on the plate. This was followed by incubation for 1 h, when 100 μL of streptavidin peroxidase was added to each well on the plate, which was washed as before. Next, 100 μL of 3,3′,5,5′-Tetramethylbenzidine was added to each well. The plate was covered in aluminum foil to avoid exposure of the plate to direct sunlight and incubated for 30 min. The reaction was stopped by adding 100 μL of stop solution. The ELISA plate was read within 30 min of adding the stop solution at 450 nm using a plate reader. Interpretation of results was performed by first obtaining a good curve for the standards in Excel. The mean absorbance value (OD) for each sample was inserted in logarithmic scale to obtain the concentration. In the graph, the OD values were plotted on the y-axis and concentrations on the x-axis.

### Statistical analysis

The data were double-entered into the questionnaire and Microsoft Excel 2010 (Microsoft Corp., Redmond, WA, USA) and statistical analysis was performed using STATA statistical software version 14 (STATA Corp., TX, USA). GraphPad Prism version 9.10 (221) was used to create the figures. Continuous variables that were non-parametric were expressed as medians and IQR. Categorical and continuous variables were analysed using χ^2^ and Wilcoxon rank-sum tests, respectively. The significance of the differences between vitamin D deficiency and insufficiency was assessed using the Kruskal–Wallis test and the relationship between vitamin D and LL-37 was analysed with Spearman's pairwise correlation. The multivariate regression analysis was modelled to predict a healthy status. p<0.05 was considered statistically significant.

## Results

Table 1 shows the demographics and characteristics of 72 patients with active TB (40 HIV-positive) and 62 healthy controls (13 HIV-positive). Nobody from either group was exposed to vitamin D supplementation. The two populations did not differ in age, gender or residence. They differed in employment status significantly.

**Table 1.** Demographic and clinical characteristics

Variable	TB cases (n=72)	Healthy contacts (n=61)	p
Gender			
Male	51 (70.8%)	36 (58.1%)	
Female	21 (29.2%)	26 (41.9%)	0.122
Age, y (median, IQR)	33 (27–38)	36.5 (28–48)	0.061
Residence			
Low density	20 (27.4%)	15 (24.2%)	
High density	52 (72.6%)	47 (75.8%)	0.672
BMI, kg/m^2^ (median, IQR)	20.4 (18.3–22.4)	21.8 (19.0–25.8)	0.011
HIV status			
Positive	40 (55.6%)	13 (21.0%)	
Negative	32 (44.4%)	49 (79.0%)	<0.001
CD4 count, cell/mm^3^ (median, IQR)	450 (232–588)	707 (497–863)	<0.001
CD4 count (HIV Negative), cell/mm^3^ (median, IQR)	556.5 (465–702.5)	754 (623–875)	<0.002
CD4 count (HIV Positive), cell/mm^3^ (median, IQR)	319 (157–450)	391.5 (266.5–579.5)	0.191
Site of infection			
Pulmonary	69 (95.8%)	–	<0.0001
Extra-pulmonary	3 (4.2%)	–	
Kernofsky score			
≤70	12 (16.7%)	–	
>70	60 (83.3%)	–	<0.0001
Episodes of TB			
First	53 (73.1%)	–	
Second or third	19 (26.9%)	–	<0.0001
Employment status			
Unemployed	65 (90.6%)	40 (64.5%)	
Employed	7 (9.4%)	22 (35.4.5%)	0.006
Total vitamin D, ng/ml (median, IQR)	28.7 (19.88–38.64)	40.8 (31.2–49.44)	<0.001
Vitamin D status			
Deficiency	14 (19.4%)	1 (1.6%)	
Insufficiency	18 (25%)	12 (19.7%)	0.002
Normal	40 (55.6%)	48 (78.7%)	
LL-37, ng/ml (median, IQR)	1.87 (1.43–13.75)	6.73 (3.95–11.75)	0.0149

Abbreviation: NA, not applicable.

**Figure 2.**
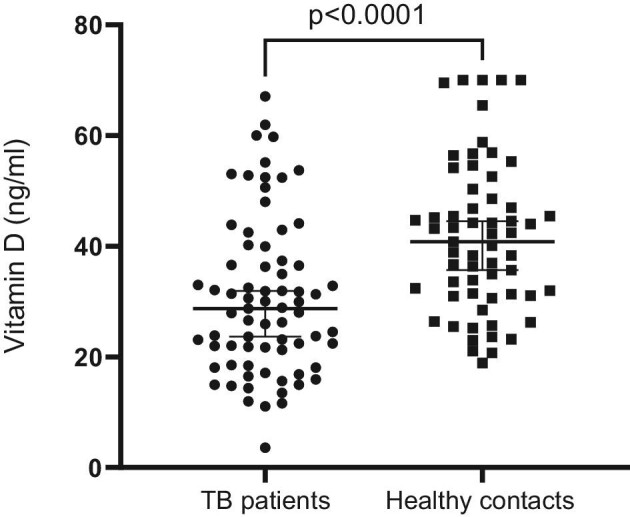
Vitamin D levels in patients and healthy contacts.

Figure 2 shows a comparison of vitamin D levels between TB patients and healthy contacts.

**Figure 3.**
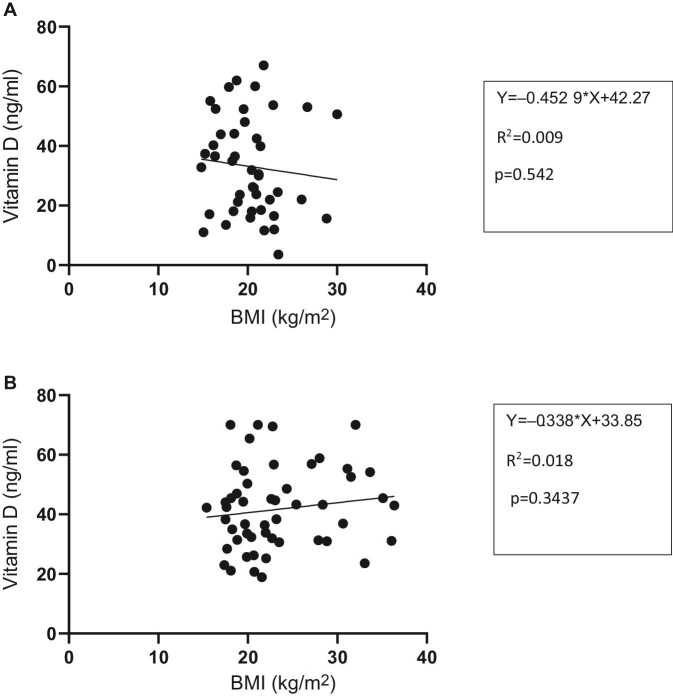
(A) Relationship between BMI and vitamin D levels in patients with TB. (B) Relationship between BMI and vitamin D levels in healthy contacts.

Figure 3A shows a negative correlation of body mass index (BMI) with vitamin D in TB patients. As the BMI increases there is a corresponding decrease in vitamin D. The observation is not significant (R^2^=0.009 [95% CI –1.933 to 1.027], p=0.5402, Y=–0.4529*X+42.27).

Figure 3B shows a positive correlation of BMI with vitamin D in TB patients. As the BMI increases there is a corresponding increase in vitamin D. The observation is not significant (R^2^=0.018 [95% CI –0.144 to 0.41], p=0.3437, Y=–0.338*X+33.85).

### LL 37 levels in TB patients and healthy contacts

**Figure 4.**
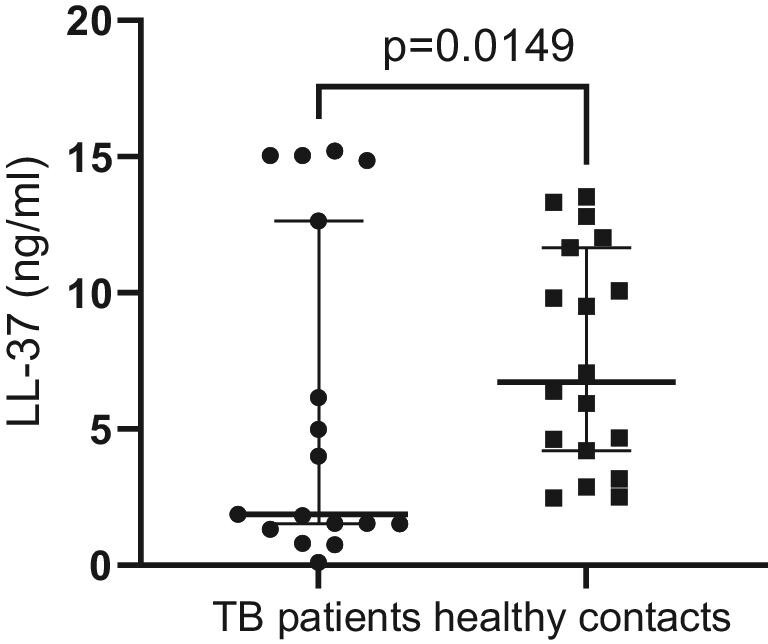
Comparison of LL 37 in TB patients vs healthy contacts.

Figure 4 shows a comparison of LL 37 in TB patients vs healthy contacts. The result shows a higher production of LL 37 in healthy contacts.

**Figure 5.**
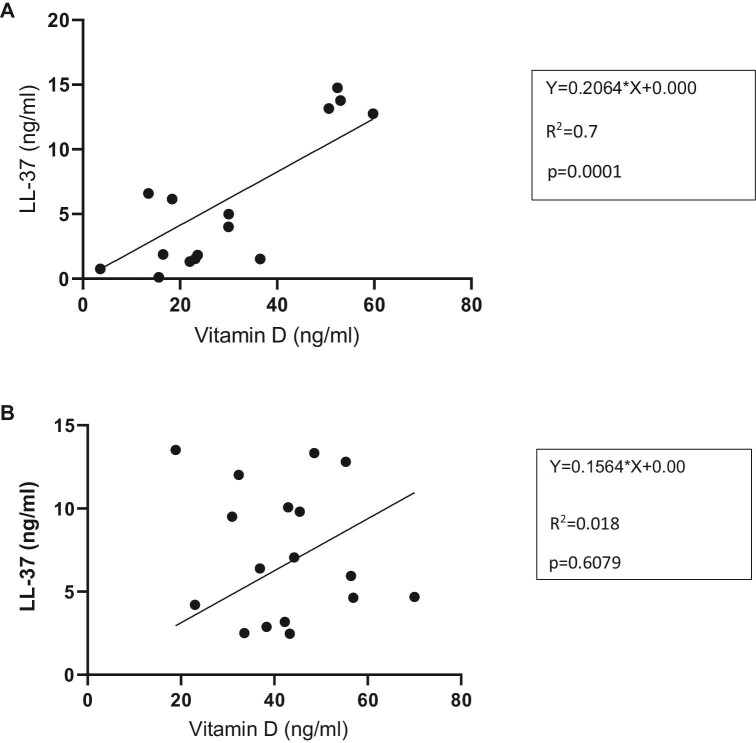
(A) Relationship between vitamin D and LL-37 in TB patients. (B) Relationship between vitamin D and LL-37 in healthy contacts.

Figure 5A shows a significant relationship between total vitamin D with LL-37 in TB patients. This may indicate that vitamin D has a stimulatory effect on the production of LL-37 (R^2^=0.7 [95% CI 0.566 to 0.944], p=0.0001).

Figure 5B shows no linear relationship between vitamin D and LL-37 in healthy contacts (R^2^=0.018 [95% CI –0.58 to 0.37], p=0.6079).

## Discussion

There are several notable findings from our study.

 First, the levels of vitamin D in newly diagnosed patients with active TB were significantly decreased. Healthy contacts, unlike TB patients, had predominantly normal levels of vitamin D. This supports previous observations from Ethiopia, South Africa and several Asian and European countries.^5,6,13^

 Second, lower levels of LL-37 were also observed in patients with active TB.

 Third, a significant linear correlation between vitamin D and LL-37 was observed in TB patients compared with healthy controls. A significant linear relationship in the control group was not seen. Fourth, in both groups, vitamin D levels were not distinguishable by HIV status. Fifth, after adjusting for confounders, a normal vitamin D and a normal CD4 count are independently protective against TB (see table 2). The mechanism for the protective effect of vitamin D is that it is essential in the production of LL-37, a pleiotropic peptide by the macrophages. Vitamin D, indirectly through LL-37, enhances the autophagy of *M. tuberculosis*.^14^

**Table 2.** Factors protective against active TB

Characteristic	Crude OR	95% CI	p	Adjusted OR	95% CI	p
Gender						
Female	1			1		
Male	0.62	0.30 to 1.27	0.191	0.40	0.54 to 0.42	0.062
Age (y)	1.03	1.00 to 1.06	**0.028**	1.03	0.99 to 1.07	0.070
Residence						
Low density	1			1		
High density	1.16	0.53 to 2.5	0.713	1.65	0.57 to 4.8	0.132
Employment status						
Unemployed	1			1		
Employed	3.4	1.36 to 8.51	**0.009**	2.31	0.78 to 69.0	0.132
HIV status						
Positive	1			1		
Negative	4.3	2.12 to 9.15	**0.000**	0.39	0.14 to 1.08	0.071
CD4 count						
≤499 cell$/$mm^3^	1			1		
500–1400 cell$/$mm^3^	3.99	1.84 to 8.66	**0.000**	2.92	1.13 to 7.6	**0.027**
Vitamin D						
Insufficiency	1			1		
Normal	4.07	1.91 to 8.65	**0.001**	**4.06**	1.68 to 9.81	**0.002**

Bolded p values are statistically significant. Other values which are not p values should not be in bold.

There are a paucity of data on the relationship of LL-37 and vitamin D in TB patients. The findings in this study support other work research that has described the relationship between vitamin D and LL-37, predominantly in people living with HIV. Tangpricha et al. found a positive correlation of LL 37 and vitamin D in children and adults living with HIV.^15^ Honda et al. evaluated the relationship of LL-37 with vitamin D in patients with HIV-1 and found a corresponding relationship between LL-37 and vitamin D.^16^ One plausible explanation for the observation regarding the relationship of vitamin D and LL-37 in healthy contacts is that they had a higher level of vitamin D and therefore produced higher levels of LL-37 within the normal range. The threshold for the relationship remains to be determined.

Males predominated in our study group. This mirrors the general observation in our routine TB programmes, which show that more males than females contract TB.^17^ The two study populations were similar in socioeconomic characteristics. Most were from a low socioeconomic setting, measured here by employment status and residence as surrogates. This illustrates that the patients were sampled from a similar population. The question arises of why then is there a variation in vitamin D status if they are from a homogenous social setting? Genetic susceptibility to vitamin D deficiency has been suggested by Pilarski et al.,^9^ and Sita-Lumsden et al.^18^ The genetic variation may explain our finding. We advocate further exploration of this observation.

Patients with TB had significantly lower BMI compared with healthy contacts and this was not an unexpected finding, because TB is known to cause wasting.^19^ We did not establish any relationship between vitamin D and any level of BMI in either study population. It is known that obesity is associated with low vitamin D. There is a functional deficiency due to reduced bioavailability brought about by sequestration of vitamin D in adipose tissue.^19-21^ The absence of obese participants in both groups is a plausible explanation for our findings.^21^

One of the baseline characteristics we looked at in this study was the CD4 count for both cohorts regardless of HIV status. We noted a significant difference in the absolute CD4 count between the two groups: TB patients had a lower CD4 count. A significant proportion of TB patients had a CD4 count below the normal threshold. This observation is supported by the findings of Ezeamama et al.^22^ and Aziza et al.,^23^ who, in a longitudinal study, established that hypovitaminosis D impairs CD4 recovery, despite antiretroviral therapy. This too requires further exploration.

The first limitation of the current study is that it did not focus on establishing the risk factors for vitamin D and other factors that influence LL-37 production. The second limitation is the non-availability of previous results for vitamin D and LL-37 levels to enable time point comparisons to derive cause and effect.

### Conclusions

The current study shows significantly lower levels of vitamin D and LL-37 in adults with newly diagnosed active TB. The study findings support the assumption that hypovitaminosis D potentiates susceptibility to TB. This relationship should be investigated further in a large-cohort longitudinal study. We advocate for longitudinal studies across various geographical regions to accurately define the roles of vitamin D and LL-37 on TB treatment and prevention.

## Data Availability

All the data from this study has been presented in the manuscript.
